# Strangling Congenital Constriction Ring Band of the Forearm with Fracture: A Rare Case Report

**DOI:** 10.7759/cureus.4189

**Published:** 2019-03-06

**Authors:** Stavros Angelis, Georgios Vynichakis, Angelos Trellopoulos, Hristos Mirtsios, John Ν Michelarakis

**Affiliations:** 1 Orthopaedics, General Hospital Hellenic Red Cross Korgialenio - Benakio, Athens, GRC; 2 Orthopaedics, General Hospital of Piraeus Tzaneio, Piraeus, GRC; 3 Orthopedics, General Children’s Hospital “Panagiotis & Aglaia Kyriakou”, Athens, GRC

**Keywords:** constriction ring syndrome, annular defect, amniotic band

## Abstract

Congenital constriction ring syndrome (CCRS) is a well-described pathological entity that is caused by fibrous bands that entrap parts of the fetus. The manifestations of this syndrome may vary a lot. We present a case of an almost intrauterine amputation of a fetus’s upper limb. Our case is infrequent because the constriction band caused a fracture of the fetus's forearm during pregnancy. Both the band and the fracture resulted in ischemia to the hand and a salvage procedure was applied after birth.

Not many authors have reported fractures due to constriction ring bands and even less have reported fractures of the upper limb. A literature review of this rare entity was conducted.

## Introduction

The etiology of congenital constriction ring syndrome (CCRS) is unknown and many theories have been proposed. The three most commonly referred to in the literature are the intrinsic theory, the theory of spontaneous rupture of amnion early in the second trimester of pregnancy, and the theory of intrauterine trauma [1].

CCRS may present with a wide range of pathology. This depends on the site of the band and the proportion of constriction that is caused. Most bands are wrapped around distal extremities, usually fingers and toes. The rest of the limbs are less commonly affected. Although most patients are only superficially affected, both neurovascular and functional damage are frequently reported. So the syndrome may be manifested with amputations, constriction bands, and pseudosyndactylism. The syndrome may also present with multiple craniofacial, visceral, and trunk anomalies.

By reviewing the literature, we came across only a few reports of bone defects. This dramatically decreases when the forearm is involved. Only a few limbs have been reported to be fractured. Most limbs that appear with osseous defects are thought to be pseudarthroses and not a recent fracture. To our knowledge, there has never been a report of a fracture of the forearm of a neonate due to an amniotic band.

## Case presentation

A 31 weeks preterm male was born by cesarian section to a 30-year-old mother. His birth weight was 1080 gm and the circumference of his head was 27 cm. Apgar scores were 6 (1 min), 5 (5 min), and 5 (10 min). His left hand was swollen due to a constriction band and, therefore, put in an elevated position. Due to respiratory distress syndrome (RDS), non-invasive positive pressure ventilation (NIPPV) was applied for two days. Also renal failure with anuria and ischemic encephalopathy were diagnosed during the next few days. The neonate's general clinical condition was critical.

Prenatal history included twin-to-twin transfusion syndrome. At the 17th week of gestation, there was an attempt to separate the fetuses, but two days later, one of them was diagnosed dead. Three days before the delivery, the fetus was diagnosed with a constriction band around its left forearm.

**Figure 1:**
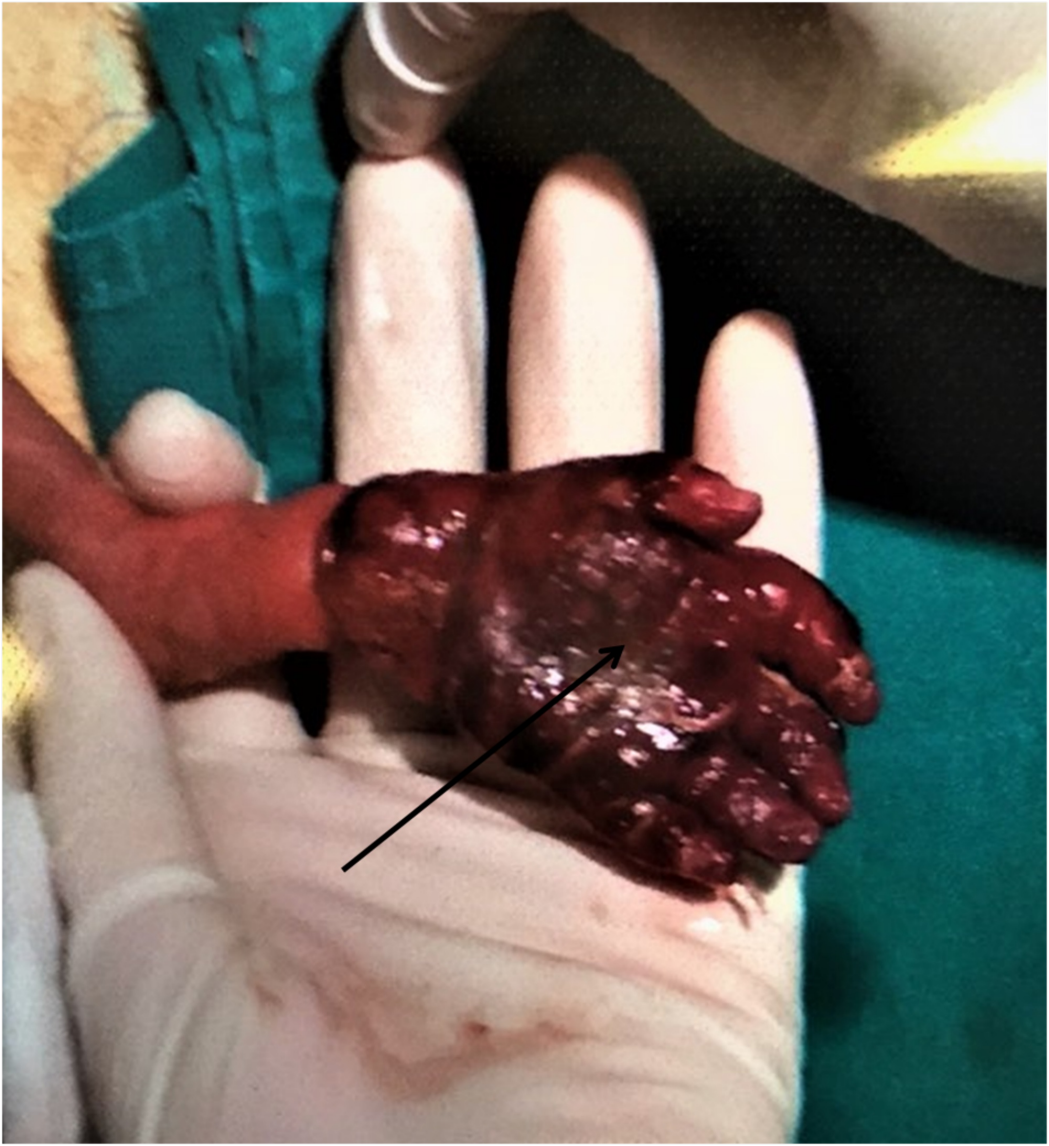
Severe swelling and vascular compromise of the hand, distal to the amniotic band.

**Figure 2:**
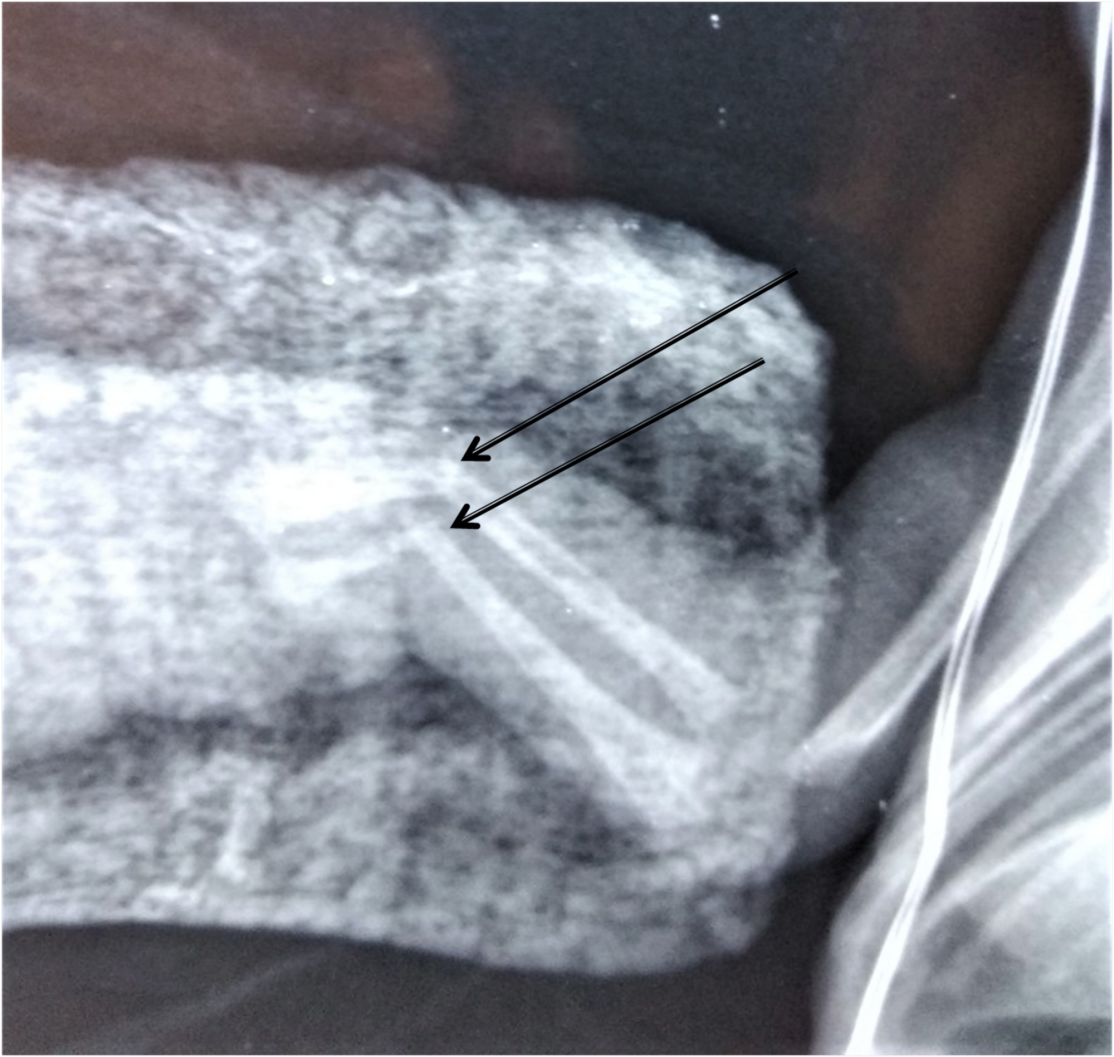
Fracture of the distal third of the radius and ulna at the site of the amniotic band.

The neonate was transferred to our institution’s neonatal intensive care unit on the second day after birth. A constriction band around the distal third of the forearm was noticed. Severe swelling and vascular compromise of the hand were evident (Figure 1). Capillary refill time at the fingers was delayed and radial pulse was barely palpable. The hand and fingers were tense and not easily compressible. The fingers did not show any signs of spontaneous movement. Radiographs of the left forearm revealed a displaced fracture of the radius and ulna at the level of the band on the distal third of the forearm (Figure 2). Abnormal motion was detected at the site of the forearm defect.

**Figure 3:**
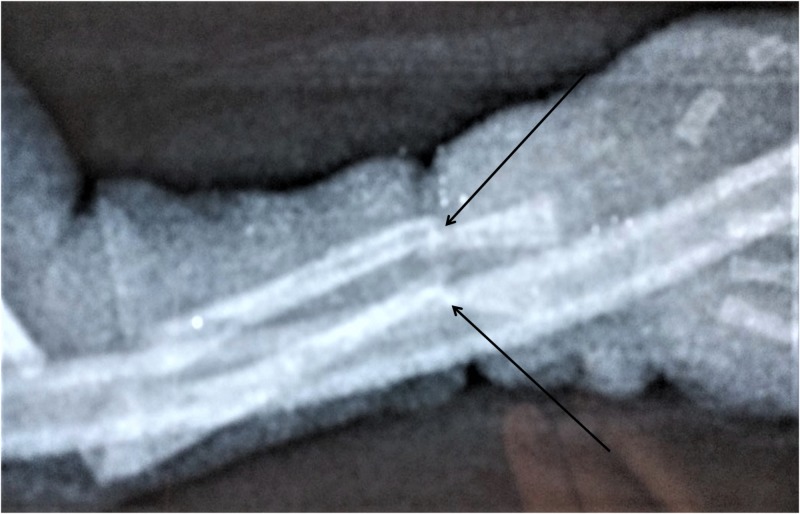
Radiograph of the fracture site after reduction.

**Figure 4:**
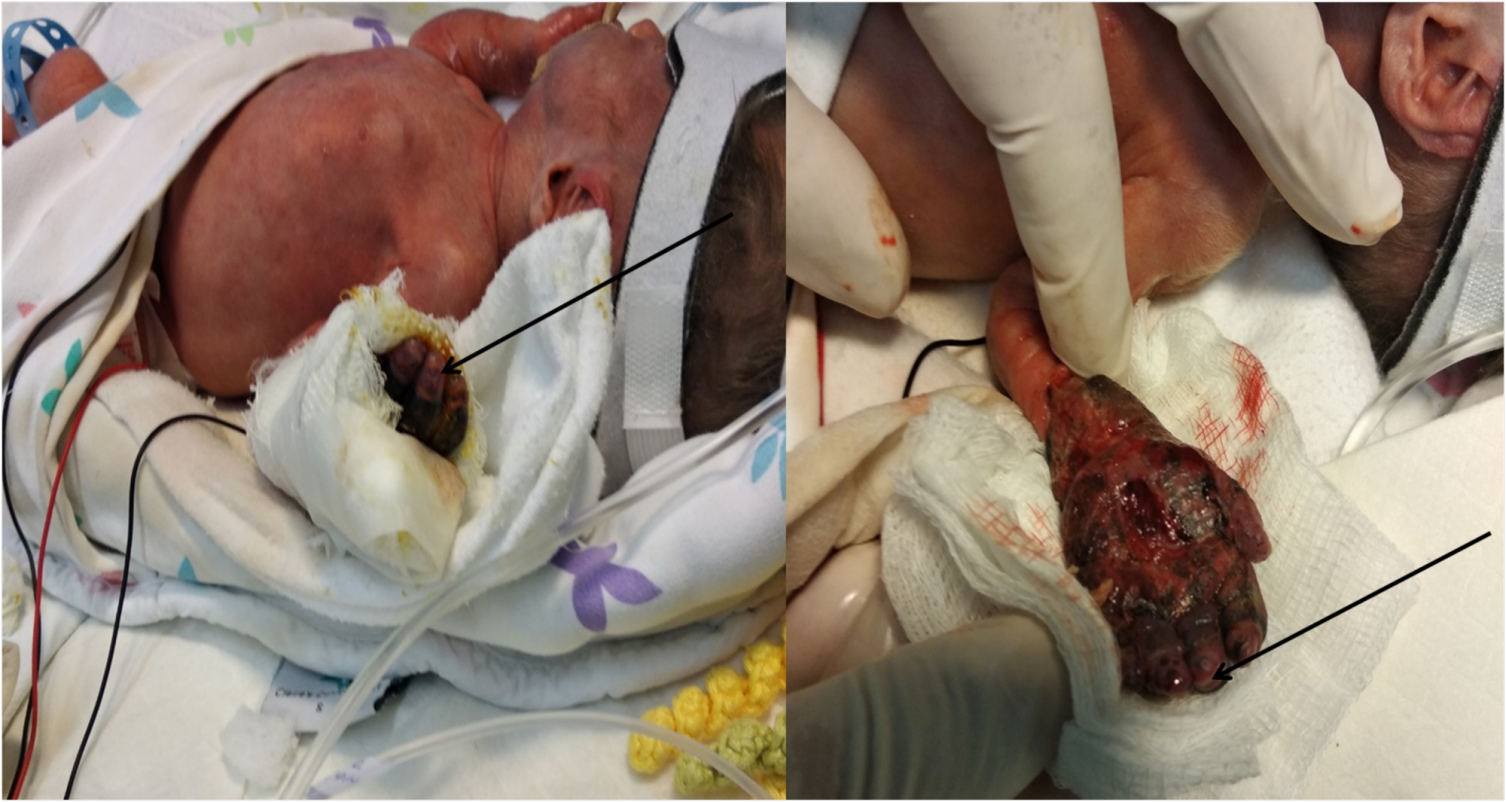
Compartment syndrome was gradually alleviated. Capillary refill and normal color of the fingertips (arrows) became evident during the next few days.

We decided to perform a reduction of the fracture and release the band. Under anesthesia and sterile preparation, a 5 mm longitudinal incision with an 11 blade scalpel over the dorsal part of the band was performed. Due to the swelling of the hand, a second 5 mm longitudinal incision was made dorsally in order to decompress it. Wounds were left open. Finally, the fracture was reduced and immobilized with a palmar cast in order to decompress the compartment syndrome (Figure 3). The hand was kept elevated. Daily wound changes with loose non-circumferential gauze and monitoring of the hand revealed salvation of the limb. The edema gradually decreased and the hand became soft and compressible. Capillary refill at the fingertips and nail beds became evident and radial pulse was palpable (Figure 4). Unfortunately, the patient deceased on the 16th day of his life.

## Discussion

CCRS is not an uncommon pathologic entity. Most cases are of mild emergence, such as a simple cutaneous constriction around a specific part of the body, but in some cases, a more severe outcome may be present. Many cases of defects that involve craniofacial, visceral, and trunk anomalies have been reported in the literature. Limbs are affected with a wide spectrum of deformity manifestation. Osseous anomalies of the limbs are not usual since the annular defect is either too deep and, therefore, causes partial or complete amputation or it is superficial and causes only cosmetic or various degrees of neurovascular compromise.

Only a few cases of limb defects have been reported and most of them concern pseudarthrosis of the tibia and fibula [2-8]. Most of these cases have been treated with debridement, decortication, and immobilization. One was treated with fasciectomy, nerve release, and pin fixation and another one was treated with external fixation and debridement [3,6]. All these articles have reported bone healing and good functional outcome [2-8].

As far as the upper limb is concerned, we have only come across two cases of pseudarthrosis of the forearm in the literature. Zionts et al. report a case of an identical twin with pseudarthrosis of the radius and ulna at the level of a congenital band around the forearm and a congenital amputation of the opposite forearm. This was treated by simple cast immobilization, and bone healing was achieved. The second infant had a band around the leg that caused minimum constriction [9]. Ho et al. report a pseudarthrosis of the forearm treated with debridement, immobilization, and a good outcome [10].

Finally, we have found only one case of recent fracture due to an amniotic band in the literature. Masmoudi et al. report a fracture of the tibia and fibula that was caused in late pregnancy at the site of the annular defect due to the increasing mechanical stress on the bones related to fetal growth within the confines of the uterine cavity. They have treated their case only with immobilization and report good outcome [11].

In our case, the radiographs indicated a recent fracture since the edges of the fractures were sharp, there were no signs of sclerosis on them, and no callus formation or periosteal reaction was present. We also believe that the fracture occurred only in late pregnancy. Probably, mechanical stress was applied to the hand at its most yielding site and this lead to the fracture. Alignment of the hand was lost and this caused vascular compromise along with the vascular distress caused by the band.

## Conclusions

We have reported an unusual case of a forearm fracture at the site of an annular defect in a male neonate treated with debridement, reduction, and immobilization. Fractures due to amniotic bands are rare. Pseudarthrosis defects are more common. Osseous defects reported in the forearm are not common. Before amputation is considered, in some cases, a more conservative approach may be considered.
